# Risk factors for postoperative ischemic complications in pediatric moyamoya disease

**DOI:** 10.1186/s12883-021-02283-9

**Published:** 2021-06-22

**Authors:** Xiaofeng Deng, Peicong Ge, Rong Wang, Dong Zhang, Jizong Zhao, Yan Zhang

**Affiliations:** 1 Department of Neurosurgery, Beijing Tiantan Hospital, Capital Medical University, Beijing, 100070 China; 2 China National Clinical Research Center for Neurological Diseases, Beijing, China; 3 Center of Stroke, Beijing Institute for Brain Disorders, Beijing, China; 4 Beijing Key Laboratory of Translational Medicine for Cerebrovascular Disease, Beijing, China; 5 Savaid Medical School, University of Chinese Academy of Sciences, Beijing, China

**Keywords:** Moyamoya disease, Pediatric, Risk factors, Ischemic complications

## Abstract

**Background:**

Ischemic events are the most common postoperative complication in bypass surgery for moyamoya disease (MMD), but the risk factors for pediatric MMD remain unclear. The goal of the study was to investigate the risk factors for postoperative ischemic complications in pediatric MMD patients.

**Methods:**

We retrospectively reviewed a consecutive series of pediatric MMD cases at Beijing Tiantan Hospital, Capital Medical University from June 2010 through June 2019. Preoperative clinical variables and radiographic findings were recorded, and logistic regression analysis was carried out to identify the risk factors for postoperative ischemic events.

**Results:**

A total of 533 operations in 336 patients were included in this study. Postoperative complications occurred after 51 operations (9.6%), including 40/447 indirect bypass procedures, 9/70 direct bypass procedures, and 2/16 combined bypass procedures. Postoperative ischemic events were the most common complication and occurred in 30 patients after 31 procedures (8.9% per patient; 5.8% per operation), including 26/447 indirect bypass procedures, 4/70 direct bypass procedures, and 1/16 combined bypass procedures, and the incidence of these events did not differ significantly between indirect and non-indirect bypass (5.8% vs 5.8%; *p* = 0.999). Multivariate logistic regression analyses revealed that older age at operation (OR 1.129, 95% CI 1.011–1.260, *p* = 0.032) and posterior cerebral artery involvement (OR 2.587, 95% CI 1.030–6.496, *p* = 0.043) were significantly associated with postoperative ischemic events.

**Conclusion:**

We speculate that older age at operation and posterior cerebral artery involvement are risk factors for postoperative ischemic events in pediatric MMD patients.

## Introduction

Moyamoya disease (MMD) is an uncommon chronic cerebrovascular occlusive disorder [1]. It is characterized by progressive stenosis or occlusion in the bilateral internal carotid arteries (ICAs) or their main branches with compensatory small arterial collaterals that appear as a “puff of smoke” on angiographic findings [2]. MMD has a low incidence in Western countries, but it is the leading cause of stroke in children and adolescents in China, South Korea and Japan [3].

Although controversy remains, revascularization surgery is the primary treatment for MMD; it prevents recurrent stroke and improves the prognosis, especially in the pediatric population [4]. It has been reported that postoperative complications in surgical revascularization for MMD occur in 5.6–20.5% of patients. Among them, ischemic events are most common (1.5–11.4%) and may lead to transient neurological deterioration or permanent neurological deficits [4–7]. Therefore, prevention of postoperative ischemic complications is very important in ensuring the benefits of surgical revascularization.

Risk factors for postoperative ischemic complications in adult patients with MMD have been well-documented, while the paucity of data on pediatric MMD patients and related evidence is limited [8]. Therefore, we performed this study to investigate the risk factors for postoperative ischemic complications in pediatric MMD.

## Materials and methods

### Patient data

The Ethics Committee of Beijing Tiantan Hospital, Capital Medical University approved this study. All MMD inpatients at Beijing Tiantan Hospital from June 2010 through June 2019 were screened. The patient inclusion criteria were as follows: 1) patients diagnosed with MMD, based on digital subtraction angiography (DSA) and/or MR angiography according to the published guidelines of the Research Committee on MMD in Japan [9]; 2) patients who received surgical revascularization; and 3) patients aged less than 18 years old at the time of operation. Patients with a history of brain tumor, cranial irradiation, Down syndrome, neurofibromatosis, meningitis and sickle cell disease were excluded [1].

The patient data collected included age at operation, sex, onset symptoms, admission modified Rankin Scale (mRS) score, past medical history (including hypertension, thyroid disease, hyperlipidemia, smoking, alcohol use, diabetes and family history), surgical modalities, length of hospital stay (LOS), and mRS score at discharge. We categorized the onset symptoms into 3 main types: ischemic-type (infarction and transient ischemic attack (TIA)), hemorrhagic-type (intraventricular hemorrhage, intracerebral hemorrhage, subarachnoid hemorrhage, etc.), and nonspecific-type (headache, epilepsy, asymptomatic, etc.).

### Radiologic profiles

Collateral circulation was evaluated based on the classification criteria reported by Liu et al. [10]. Posterior collateral circulation included 3 parts and was evaluated as follows: (1) on lateral views of vertebrobasilar artery (VA) angiograms, the leptomeningeal collateral networks from the posterior cerebral artery (PCA) territory to the anterior cerebral artery (ACA) territory were given 1 point if blood supply to the cortical border zone between the ACA and PCA territory was present and 2 points if blood supply over the central sulcus via the posterior pericallosal artery we present; (2) on anteroposterior views of VA angiograms, the leptomeningeal collateral networks from the anterior temporal branch of the PCA to the middle cerebral artery (MCA) territory were given 1 point if anastomoses of the anterior temporal branches of the PCA and temporal branches of the MCA were present; (3) also on anteroposterior views of VA angiograms, the leptomeningeal collateral networks from the parieto-occipital branch of the PCA to the MCA territory were given 1 point if the retrograde flow of parieto-occipital branch of the PCA extended to the M4 segment of the MCA (superficial vessels only), 2 points if the blood supply extended into the Sylvian fissure and 3 points if the blood supply extended into the occlusion within the M1 or proximal M2 segments.

Anterior collateral circulation was evaluated by Suzuki stage [2], and scores of 6 to 0 were corresponded to Suzuki stages 0 to 6, respectively.

The sum of the four abovementioned scores was calculated, and the stages of collateral circulation were categorized as follows: Grade I, a score of 0 to 4; Grade II, a score of 5 to 8; and Grade III, a score of 9 to 12.

### Surgical modalities

Indirect procedures involve placing various connective tissues on the surface of the brain to promote revascularization [4]. In our center, encephaloduroarteriosynangiosis (EDAS) and multiple burr holes (MBH) were performed. For EDAS, the branch of the superficial temporal artery (STA) was placed on the brain surface after being dissected. Intraoperative indocyanine green (ICG) fluorescence angiography was used to ensure the patency of the STA. In terms of MBHs, five to fifteen burr holes were drilled over the hypoperfusion brain areas, and the dura was opened and separated. Direct procedures involved end-to-side anastomosis of the STA to the M4 branch of the MCA. Combined bypass was defined as direct and indirect bypass were both performed on one hemisphere during one operation [11]. The immediate patency of anastomosis in direct and combined bypass was also evaluated by intraoperative ICG fluorescence angiography. Corresponding adjustments must be taken if the arteries were not patent, until patency was ensued by repeated ICG fluorescence angiography.

### Postoperative management and complications

A standardized perioperative management protocol was applied in all cases. All pediatric patients received fluid therapy and blood pressure (BP) monitoring after revascularization. For patients receiving indirect bypass, postoperative BP was controlled greater (no more than 20%) than or equal to the baseline BP. For patients with direct and combined bypass, postoperative BP was controlled to be less (no more than 20%) than or equal to the baseline BP in cases of hyperperfusion syndrome [12]. Patients received a CT scan 4 to 6 h after the procedure. Repeated CT and/or MRI scans were performed if the patient exhibited postoperative neurological deterioration. Postoperative complications were recorded during the hospital stay, including TIA, infarction, cerebral hyperperfusion syndrome, subdural effusion, intracranial infection, seizures, impaired wound healing, and subdural hematoma. Cerebral hyperperfusion syndrome was characterized by a series of neurological deficits, including a focal seizure, reversible deterioration of consciousness level with behavioral and/or aphasia, or intracerebral hemorrhage, along with absence of a definite new infarction on a brain CT scan and/or diffusion-weighted MRI. And the diagnosis was further confirmed by a CT perfusion scan [13].

### Statistical analysis

Statistical analysis was performed using SPSS (Windows version 22.0, IBM). Differences between patients with and without postoperative complications were compared by the Student’s t test for continuous variables and the chi-square test for categorical variables. The Logistic regression analysis was performed to test which variables were associated with postoperative complications. Clinical variables that achieved a *p* value < 0.10 in the univariate analysis were included in the multivariate analysis. A *p* value < 0.05 was defined as statistically significant. In addition, the statistical program R (version 4.0.2; R core team) was used to create a nomogram.

## Results

### Baseline characteristics and the incidence of postoperative complications

A total of 336 pediatric patients were enrolled in this study. The median time from onset to the first procedure was six months. Among these patients, 144 underwent one revascularization procedure, 187 underwent two operations, and 5 underwent three operations. Overall, a total of 533 procedures were performed, including 447 indirect bypasses, 70 direct bypasses, and 16 combined bypass procedures. The mean age at the time of operation was 9.6 ± 3.7 years (Table 1). Based on onset symptoms, the clinical presentation was ischemic-type in 366 cases (68.7%), hemorrhagic-type in 28 (5.3%), and nonspecific-type in 43 (26.1%). As shown in Table 1, compared with patients without postoperative complications, patients with postoperative complications tended to be older, and had more cases with PCA involvement and higher collateral circulation grades (all *p* < 0.05). In addition, patients with postoperative complications had longer hospital stays (*p* < 0.0001) and worse mRS scores at discharge (*p* < 0.0001).

**Table 1:** Baseline characteristics of hemispheres with and without postoperative complications

Characteristics	Total (*n* = 533)	Postop Complications		*P* value
		Present (*n* = 51)	Absent (*n* = 482)	
Mean age at op (yrs)	9.6 ± 3.7	10.9 ± 4.1	9.5 ± 3.6	**0.008**
Sex, female/male ratio	272/261	27/24	245/237	0.774
Clinical presentation				
Ischemic	366 (68.7)	31 (60.8)	335 (69.5)	0.429
Infarction	132 (24.8)	12 (23.5)	120 (24.9)	
TIA	234 (43.9)	19 (37.3)	215 (44.6)	
Hemorrhagic	28 (5.3)	3 (5.9)	25 (5.2)	
Nonspecific	139 (26.1)	17 (33.3)	122 (25.3)	
Headache	38 (7.1)	6 (11.8)	32 (6.6)	
Epilepsy	38 (7.1)	5 (9.8)	33 (6.8)	
Asymptomatic	63 (11.8)	6 (11.8)	57 (11.8)	
Admission mRS score				
Mean	1.4 ± 0.6	1.5 ± 0.6	1.4 ± 0.6	0.260
0–1	371 (69.6)	31 (60.8)	340 (70.5)	0.150
2–5	162 (30.4)	20 (39.2)	142 (29.5)	
Past medical history				
Family history	38 (7.1)	4 (7.8)	34 (7.1)	1.000
Hypertension	7 (1.3)	1 (2.0)	6 (1.2)	1.000
Thyroid disease	4 (0.8)	1 (2.0)	3 (0.6)	0.841
Hyperlipidemia	3 (0.6)	0 (0.0)	3 (0.6)	1.000
Smoking & Alcohol use	2 (0.4)	0 (0.0)	2 (0.4)	1.000
Diabetes	1 (0.2)	0 (0.0)	1 (0.2)	1.000
Suzuki stage^a^				
Mean	2.9 ± 0.9	3.0 ± 0.8	2.9 ± 0.9	0.238
1	12 (2.4)	0 (0.0)	12 (2.7)	0.268
2	154 (31.1)	14 (29.2)	140 (31.3)	
3	216 (43.6)	20 (41.7)	196 (43.8)	
4	96 (19.4)	12 (25.0)	84 (18.8)	
5	17 (3.4)	2 (4.2)	15 (3.4)	
PCA involvement^a^	116 (23.4)	18 (37.5)	98 (21.9)	**0.015**
Collateral circulation^a^				
Mean	7.0 ± 1.8	6.3 ± 2.1	7.0 ± 1.8	**0.019**
Grade I (0–4)	54 (10.9)	12 (25.0)	42 (9.4)	
Grade II (5–8)	342 (69.1)	26 (54.2)	316 (70.7)	
Grade III (9–12)	99 (20.0)	10 (20.8)	89 (19.9)	
ECA collateral^a^				
STA collateral	4 (0.8)	1 (2.1)	3 (0.7)	0.849
MMA collateral	197 (39.8)	21 (43.8)	176 (39.4)	0.556
OA collateral	29 (5.9)	4 (8.3)	25 (5.6)	0.656
Surgical modalities				
Indirect bypass	447 (83.9)	40 (78.4)	407 (84.4)	0.540
Direct bypass	70 (13.1)	9 (17.6)	61 (12.7)	
Combined bypass	16 (3.0)	2 (3.9)	14 (2.9)	
Mean LOS (days)	15 (13–18)	20.0 ± 8.8	15.3 ± 3.8	**0.000**
Discharge mRS score				
Mean	1.4 ± 0.6	1.7 ± 0.8	1.3 ± 0.6	**0.000**
0–1	378 (70.9)	22 (43.1)	356 (73.9)	**0.000**
2–5	155 (29.1)	29 (56.9)	126 (26.1)	

*ECA* External carotid artery, *LOS* Length of hospital stay, *MMA* Middle meningeal artery, *OA* Occipital artery, *PCA* Posterior cerebral artery, *STA* Superficial temporal artery, *TIA* Transient ischemic attacking

^a^495 hemispheres received DSA

In 533 operations, 55 postoperative complications were observed in 50 patients after 51 revascularization procedures (14.8% per patient; 9.6% per operation). Stratified by surgical modality, the number and rate of complications were as follows (Table 2): 40/447 (8.9%) for indirect bypass, 9/70 (12.9%) for direct bypass, and 2/16 (12.5%) for combined bypass (11/86 for non-indirect bypass, overall), and there was no significant difference between indirect and non-indirect bypass (8.9 vs 12.8%; *p* = 0.267).

**Table 2:** Postoperative complications stratified by surgery modalities

Complications	Total (*n* = 533)	Surgical modalities		*P* value
		IB (*n* = 447)	DB and CB (*n* = 86)	
Total	51 (9.6)	40 (8.9)	11 (12.8)	0.267
Ischemic events	31 (5.8)	26 (5.8)	5 (5.8)	0.999
Infarction	9 (1.7)	6 (1.3)	3 (3.5)	0.338
TIA	22 (4.1)	20 (4.5)	2 (2.3)	0.534
Cerebral hyperfusion syndrome	4 (0.8)	0 (0.0)	4 (4.7)	**0.001**
Subcutaneous effusion	6 (1.1)	5 (1.1)	1 (1.2)	1.000
Intracranial infection	6 (1.1)	4 (0.9)	2 (2.3)	1.000
Seizure	5 (0.9)	5 (1.1)	0 (0.0)	1.000
Impaired wound healing	3 (0.6)	2 (0.4)	1 (1.2)	0980
Subdural hematoma	1 (0.2)	0 (0.0)	1 (1.2)	0.161

*IB* Indirect bypass, *DB* Direct bypass, *CB* Combined bypass

Postoperative ischemic events (TIAs and infarctions of variable size) were the most clinically relevant complications and occurred in 30 patients after 31 procedures (8.9% per patient; 5.8% per operation), including 26/447 (5.8%) for indirect bypass, 4/70 (5.7%) for direct bypass, and 1/16 (6.25%) for combined bypass. Therefore, for indirect bypass and non-indirect bypass, the incidence of ischemic events was both 5.8% (26/447 and 5/86, Table 2). In addition, CHS was observed in 4 patients after 4 procedures (1.2% per patient; 0.8% per operation). All CHS occurred after non-indirect bypass (3 direct bypass and 1 combined bypass), and its incidence was significantly higher than that after indirect bypass (4.7 vs 0.0%, *p* = 0.001).

The other complications were as follows: 6 subcutaneous effusions (1.1% per operation, including 1 for direct bypass, 4 for EDAS, and 1 for MBH), 6 intracranial infections (1.1% per operation, including 2 for direct bypass, 3 for EDAS, and 1 for MBH), 5 seizures (0.9% per operation, all 5 for EDAS), 3 instances of impaired wound healing (0.6% per operation, including 1 for combined bypass, and 2 for EDAS), and 1 subdural hematoma (0.2% per operation, 1 for direct bypass) (some patients had two or more complications after one operation). No patient died due to these complications.

### Risk factors for postoperative ischemic events

Among the 31 postoperative ischemic events, there were 22 TIAs (one patient had TIAs after both procedures) and 9 cerebral infarctions. Notably, all 21 patients who suffered TIAs made a full recovery by 3 months after the operation. Among the 9 patients who suffered cerebral infarction, 4 had mild disability (mRS 2), 4 had moderate disability (mRS 3), and 1 had an unfavorable outcome (mRS 4). Univariate logistic regression analyses of the preoperative clinical variables showed that older age at operation (OR 1.093, 95% CI 0.991—1.206, *p* = 0.074) and PCA involvement (OR 1.985, 95% CI 0.916—4.303, *p* = 0.082) may be associated with postoperative ischemic events (Table 3). After adjusting for all potential covariables, including sex, Suzuki stage, middle meningeal artery (MMA) collateral, occipital artery (OA) collateral, and surgical modalities, multivariate logistic regression analyses revealed that older age at operation (OR 1.129, 95% CI 1.011—1.260, *p* = 0.032) and PCA involvement (OR 2.587, 95% CI 1.030 – 6.496, *p* = 0.043) were predictors of postoperative ischemic complications.

**Table 3:** Logistic regression analysis for postoperative ischemic events

Characteristics	Postop Ischemic Events		*P* value		OR (95% CI)
	Present (*n* = 31)	Absent (*n* = 502)	Uni	Multi	
Mean age at op (yrs)	10.8 ± 4.0	9.5 ± 3.7	0.074	**0.032**	1.129 (1.011–1.260)
Sex, female/male ratio	17/14	255/247	0.662	0.679	1.174 (0.550–2.504)
Ischemic presentation	19 (61.3)	347 (69.1)	0.707		
Admission mRS score					
Mean	1.4 ± 0.6	1.4 ± 0.6	0.789		
0–1	20 (64.5)	351 (30.1)			
2–5	11 (35.5)	151 (69.9)			
Past medical history					
Family history	2 (6.5)	36 (7.2)	0.880		
Hypertension	1 (3.2)	6 (1.2)	0.355		
Thyroid disease	1 (3.2)	3 (0.6)	0.143		
Hyperlipidemia	0 (0.0)	3 (0.6)	0.999		
Smoking & Alcohol use	0 (0.0)	2 (0.4)	0.999		
Diabetes	0 (0.0)	1 (0.2)	1.000		
Suzuki stage^a^					
Mean	3.0 ± 0.9	2.9 ± 0.9	0.522	0.796	1.061 (0.677–1.662)
1	0 (0.0)	12 (2.6)			
2	10 (33.3)	144 (31.0)			
3	11 (36.7)	205 (44.1)			
4	8 (26.7)	88 (18.9)			
5	1 (3.3)	16 (3.4)			
PCA involvement^a^	11 (36.7)	105 (22.6)	0.082	**0.043**	2.587 (1.030–6.496)
Collateral circulation^a^					
Mean	6.6 ± 2.1	7.0 ± 1.8	0.276		
Grade I (0–4)	6 (20.0)	48 (10.3)			
Grade II (5–8)	16 (53.3)	326 (70.1)			
Grade III (9–12)	8 (26.7)	91 (19.6)			
ECA collateral^a^					
STA collateral	0 (0.0)	4 (0.9)	0.999		
MMA collateral	11 (36.7)	186 (40.0)	0.718	0.154	0.533 (0.224–1.267)
OA collateral	3 (10.0)	26 (5.6)	0.326	0.659	1.365 (0.343–5.433)
Surgical modalities					
Indirect bypass	26 (83.9)	421 (83.9)	0.942	0.739	1.435 (0.171–12.013)
Direct bypass	4 (12.9)	66 (13.1)	0.934	0.985	0.979 (0.099–9.718)
Combined bypass	1 (3.2)	15 (3.0)			

*ECA* External carotid artery, *MMA* Middle meningeal artery, *OA* Occipital artery, *PCA* Posterior cerebral artery, *STA* Superficial temporal artery

^a^495 hemispheres received DSA

**Fig. 1:**
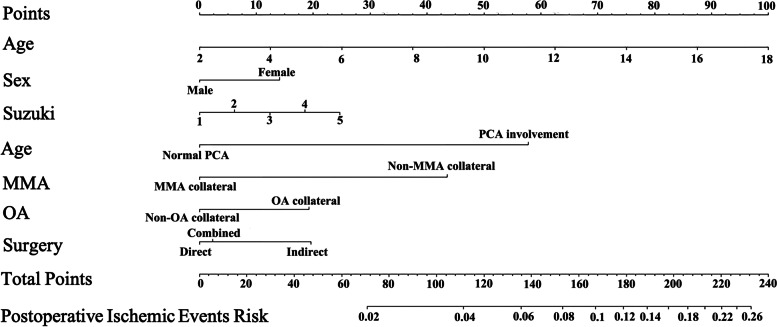
Nomogram for assessing postoperative ischemic event risk in pediatric MMD

To assess the probability of stroke after revascularization surgery in pediatric MMD patients, a nomogram was constructed based on the risk factors identified by the multivariable analysis (Fig. 1).

## Discussion

Our study showed that the incidence of the postoperative complication was 9.6% (51/533) in pediatric MMD patients. The most common complication was ischemic events (5.8%, 31/533). Older age at operation and PCA involvement were identified as predictors of postoperative ischemic events. Moreover, postoperative complications were significantly related to longer hospital stays and worse outcome at discharge.

Surgical revascularization is an effective treatment for MMD in preventing recurrent stroke and to improving prognosis, especially in the pediatric population [4, 14]. Previous studies have demonstrated that indirect, direct, and combined bypass techniques are all effective options for revascularization in pediatric MMD, but each procedure has its own advantages and disadvantages [5, 14–16]. For example, according to a meta-analysis, the estimated stroke rate was higher in indirect bypass (9.0%) than in direct bypass (4.5%) and combined bypass (6.0%) [14]. Our recent study showed that during the short term after surgery, direct bypass might be superior to indirect bypass in preventing recurrent ischemic strokes [15]. In addition, it usually takes months to improve cerebral blood flow after indirect bypass because neovascularization from connective tissue is not immediate [4]. However, direct and combined bypass are technically difficult, requiring more training and experience, especially in pediatric patients, due to the small caliber of the donor and recipient arteries [17]. Moreover, it has been reported that postoperative CHS often develops after combined and direct bypass surgery [13, 18, 19]. In the current study, all instances of CHS occurred in direct and combined bypass, and the incidence was significantly higher than that in indirect bypass.

Postoperative ischemic events may lead to potential neurological deterioration or permanent neurological deficits [8, 14, 20]. Ha et al. reported that the incidence of postoperative infarctions was 12% per patient and 6% per operation after 1283 operations; surgery-related infarctions were the most common complications and were closely related to an unfavorable outcome [7]. Kim et al. reported that that the incidence of the postoperative infarctions was 13% per patient and 6% per operation after 845 operations [5]. Bao et al. reported that the incidence of postoperative TIA or infarctions was 4.8% per patient and 2.7% per operation after 512 operations [6]. In our study, the incidence of postoperative ischemic events was 8.9% per patient and 5.8% per operation after 533 procedures, which was consistent with previous reports.

Regarding postoperative complications in pediatric MMD, there have only been a few reports with a small series of patients enrolled, and the evidence on risk factors is limited [12, 21, 22]. Muraoka et al. enrolled 58 revascularizations in 37 children and found that preoperative cerebral infarctions, younger age, higher Suzuki grade, and PCA involvement were associated with postoperative complications [21]. In this study, we also found that age at operation and PCA involvement were predictors of postoperative ischemic events. The difference is that we found that older age at operation is a risk factor for postoperative ischemic events, and we speculate that this difference might be attributed to different populations and different numbers of cases. Moreover, the leptomeningeal system from the PCA plays an important role against ischemic and hemorrhagic stroke in MMD [10, 23]. PCA involvement may suggest failed compensatory collateralization. When undergoing a surgical revascularization, patients with PCA involvement may be more vulnerable than patients without PCA involvement, and have a higher incidence of postoperative ischemic events. Therefore, patients with older age at operation or PCA involvement need special attention in perioperative management.

Our previous study reported that advanced Suzuki stage and preoperative ischemic presentation weres associated with postoperative ischemic complications in adult MMD patients [8]. However, the present study showed that Suzuki stage and ischemic presentation were not risk factors in the pediatric population. One possible explanation is that most pediatric patients receive indirect bypass, but many adult patients in our institute receive direct and combined bypass. For patients with an advanced Suzuki stage, direct and combined bypass might be difficult due to the small caliber of the recipient artery [4], which may increase the risk of complications. Moreover, the majority of pediatric patients presented with ischemia, and the proportion of hemorrhagic-type patients was significantly lower in the pediatric population (5.3% in the current study) than in the adult population (32.1% in our previous study) [24]. This may explain why ischemia at presentation was not associated with postoperative ischemic events in pediatric MMD patients.

There have recently been some studies on perioperative management to prevent postoperative ischemic events in pediatric MMD patients. For example, Honjo et al. tried to use dexmedetomidine to prevent crying after surgical revascularization in pediatric patients [25]. The study of Lee et al.showed that dysfunctional intraoperative BP autoregulation may increase the risk of TIA in pediatric patients [12, 26]. Perioperative care with BP control combined with the administration of aspirin may reduce the potential risk of postoperative ischemic complications [16]. These studies are interesting and might be useful to prevent postoperative ischemic events in pediatric patients.

The present study has some limitations. First, this study was retrospectively carried out in a single center, and although the number of cases was large, selection bias may exist. Second, not all patients underwent DSA examination, and we could not evaluate preoperative collateral circulation in all patients. Third, the number of patients who received direct and combined bypass was small. Fourth, RNF213 was not sequenced, and the effect of the variants on postoperative ischemic events remains unknown. Further prospective studies are still needed to determine the predictors of postoperative ischemic complications in pediatric MMD patients.

## Conclusion

Older age at operation and posterior cerebral artery involvement are risk factors for postoperative ischemic events in pediatric MMD patients.

## Data Availability

The datasets supporting the conclusions of this study are available from the corresponding author on reasonable request.

## Abbreviations

ACA: Anterior cerebral artery
CHS: Cerebral hyperperfusion syndrome
DSA: Digital subtraction angiography
ECA: External carotid artery
EDAS: Encephaloduroarteriosynangiosis
LOS: Length of hospital stays
MBH: Multiple burr hole
MCA: Middle cerebral artery
MMA: Middle meningeal artery
MMD: Moyamoya disease
mRS: Modified rankin scale
PCA: Posterior cerebral artery
STA: Superficial temporal artery
TIA: Transient ischemic attack
VA: Vertebrobasilar artery
